# Determination of 18 Intact Glucosinolates in *Brassicaceae* Vegetables by UHPLC-MS/MS: Comparing Tissue Disruption Methods for Sample Preparation

**DOI:** 10.3390/molecules27010231

**Published:** 2021-12-30

**Authors:** Xiaolu Yu, Hongju He, Xuezhi Zhao, Guangmin Liu, Liping Hu, Bing Cheng, Yaqin Wang

**Affiliations:** Institute of Agri-Food Processing and Nutrition, Beijing Vegetable Research Center, Beijing Academy of Agriculture and Forestry Sciences, Beijing 100097, China; yuxiaolu1015@foxmail.com (X.Y.); hehongju@iapn.org.cn (H.H.); lysozyme@foxmail.com (X.Z.); liuguangmin@iapn.org.cn (G.L.); huliping@iapn.org.cn (L.H.); chengbing@iapn.org.cn (B.C.)

**Keywords:** intact glucosinolates, *Brassicaceae* vegetables, triple quadrupole mass spectrometry, tissue disruption

## Abstract

Glucosinolates (GSLs) are important precursor compounds with anticancer activities in *Brassicaceae* vegetables and are readily hydrolyzed by myrosinase. Given the diversity of these species, establishing an accurate and universal method to quantify intact GSLs in different plant tissues is necessary. Here, we compared and optimized three tissue disruption methods for sample preparation. After microwave treatment for 90 s, 13 GSLs in homogenized Chinese cabbage samples were recovered at 73–124%. However, a limitation of this method was that different tissues could not be processed under the same microwave conditions. Regarding universality, GSLs in *Brassicaceae* vegetables could be extracted from freeze-dried sample powder with 70% methanol (*v*/*v*) or frozen-fresh sample powder with 80% methanol (*v*/*v*). Moreover, heating extraction is necessary for GSLs extracted from frozen-fresh sample powder. Average recoveries of the two optimized methods were 74–119% with relative standard deviations ≤ 15%, with the limits of quantification 5.72–17.40 nmol/g dry weight and 0.80–1.43 nmol/g fresh weight, respectively. Notably, the method for analyzing intact GSLs was more efficient than that for desulfo-GSLs regarding operational complexity, detection speed and quantification accuracy. The developed method was applied to identify the characteristic GSLs in 15 *Brassicaceae* vegetables, providing a foundation for further research on GSLs.

## 1. Introduction

Glucosinolates (GSLs) are a group of sulfur-containing secondary metabolites that are abundant mainly in *Brassicaceae* species, such as broccoli, mustard, cabbage and radish [1]. They consist of a sulfur-bound *β*-d-glucopyranose/*β*-thioglucose moiety, a sulfonated oxime and a side chain derived from amino acids. According to differences in the side chain, they are classified as aliphatic, indolic and aromatic GSLs. GSLs contribute to the unique flavor of *Brassicaceae* vegetables, including their bitter/pungent taste, and play an essential role in defending the plants against attacks by harmful insects or pathogens [2,3]. Moreover, GSLs and their breakdown products have potential anti-inflammatory and anticancer activities [4,5]. Considering their health benefits to humans, a great deal of research has been aimed at screening GSL-enriched vegetables, regulating GSL metabolism, increasing the content of GSLs and isolating and purifying GSLs. For these reasons, it is necessary to establish a reliable, accurate and validated analytical method for the quantification of GSLs [6,7,8].

The first step of sample preparation for GSL quantification is plant tissue disruption. However, GSLs can be hydrolyzed by myrosinase when the cell walls of these plants are damaged in the presence of water [9]. Therefore, it is necessary to inhibit the activity of endogenous myrosinase in the process of plant tissue disruption. In most studies, freeze-dried samples of large plant tissues were ground at room temperature to obtain homogeneous and representative samplings that could be stored for a long time. However, this method is an energy-intensive and costly process that requires long drying time. Grinding the frozen-fresh samples with liquid nitrogen or disrupting them in 80% cold methanol (precooled to −20 °C) could also inhibit the enzyme activity and ensure the extraction efficiency of GSLs [10,11]. However, with this method, it is difficult to obtain representative samples for large plant tissues. Microwave-assisted extraction, which can increase the temperature rapidly and inactivate the enzyme, was also used to extract bioactive compounds from cabbages [12,13]. More importantly, microwave treatment could preserve the GSL content in red cabbage under specific microwave time/energy input conditions [14]. It has not been reported that microwaves are used to inactivate myrosinase during the plant tissue disruption process. In the next step, GSLs are usually extracted with boiling water or aqueous methanol to ensure the denaturation of myrosinase. Some studies reported that aqueous methanol at room temperature could also extract GSLs from plant tissue [10,15]. It remains to be verified whether heating is a necessary step to extract GSLs from samples when using different plant tissue disruption methods.

GSLs are most commonly detected by reversed-phase high-performance liquid chromatography (HPLC) with UV absorption spectroscopy [16,17], in which all strong-acid residues should be removed (desulfation). Desulfation is achieved by enzymatic hydrolysis with sulfatase enzyme to remove the SO_3_^−^ group from intact GSLs, with more than 12 h of incubation required for sufficient reaction [18,19]. Enzyme concentration, pH and reaction time might affect the difficulty in interpreting the results for individual GSLs [20]. The variation in myrosinase activity may lead to the low applicability of the method for different plants [21]. In most cases, one GSL was used as an internal standard to quantify other GSLs, which may lead to error from the differences in instrument response to each GSL [22,23]. In addition, the qualitative analysis of individual GSLs is based on the order of elution or retention time, which is susceptible to interference from impurities or unknown GSLs, leading to biased results. Importantly, with widespread attention to more GSLs, HPLC methods have difficulty meeting the requirements for a quantitative study of trace-level GSLs due to its high detection limits. Recently, ultrahigh-performance liquid chromatography with mass spectrometry (UHPLC-MS/MS) has been used to determine intact GSLs. High-resolution mass spectrometry was used to identify and detect intact GSLs based on accurate masses and possible fragmentation patterns in the absence of available standards [15,24]. However, it is difficult to evaluate the accuracy of the method given the absence of validation in most studies. Some GSL standards were obtained following the structural identification of substantial GSLs, which are commonly found in *Brassicaceae* vegetables. With these standards, the MRM mode of UHPLC-MS/MS is the best technique for the separation and quantitation of GSLs because of its good separation efficiency, low detection limits and fast analysis speed. Therefore, this study aims to establish an accurate and universal method based on UHPLC-MS/MS to quantify intact GSLs in different *Brassicaceae* vegetables by comparing the efficacies of three tissue disruption methods for sample preparation.

## 2. Results and Discussion

### 2.1. Pretreatment Optimization

**Figure 1:**
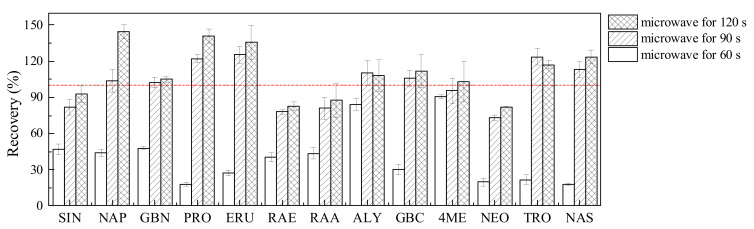
The effect of microwave treatment at different times on the recoveries of 13 GSLs in a Chinese cabbage sample. SIN, Sinigrin; NAP, Gluconapin; GBN, Glucobrassicanapin; PRO, Progoitrin; ERU, Glucoerucin; RAE, Glucoraphenin; RAA, Glucoraphanin; ALY, Glucoalyssin; GBC, Glucobrassicin; 4ME, 4-Methoxyglucobrassicin; NEO, Neoglucobrassicin; TRO, Glucotropaeolin; NAS, Gluconasturtiin.

Microwave treatment could inactivate endogenous enzymes by increasing the temperature of plant tissues rapidly [12,25]. A previous study found that myrosinase was inactivated entirely after microwave treatment at 900 W for 4.8 min [14]. However, microwave treatment may lead to the loss of glucobrassicin (GBC) since this GSL is thermally degraded at 100 °C for 10 min [10]. In our study, microwave treatment within 120 s resulted in no significant loss of 13 GSLs in standard solution (Figure S1), but it had a significant effect on GSL content in Chinese cabbage. As shown in Figure 1, the recoveries of GSLs in a Chinese cabbage sample were significantly lower than 100% after microwave treatment for 60 s, due to the insufficient inactivation of myrosinase. After microwave treatment for 90 s, the recoveries of 13 GSLs were in the range of 73–124%, indicating that this condition could inactivate myrosinase completely. The increase in the GSL recoveries with microwave treatment for 120 s might be due to the increased extractability of GSLs [14,26].

**Figure 2:**
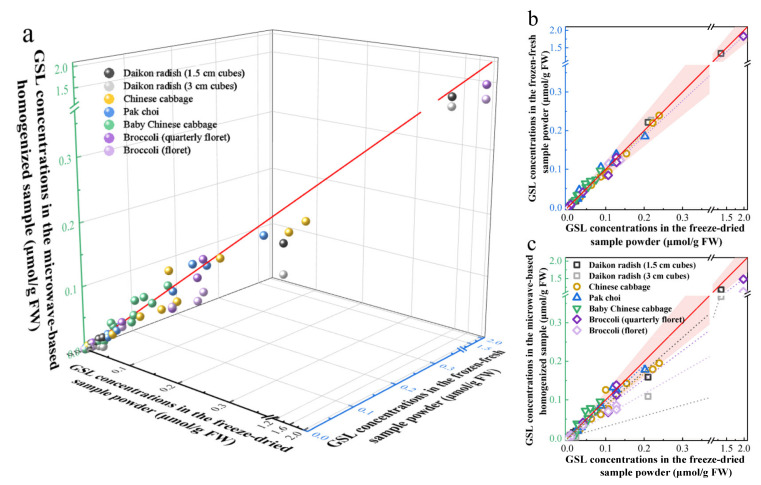
Comparison of GSL concentrations in the freeze-dried sample powder (coordinate axis in black), the frozen-fresh sample powder (coordinate axis in blue) and the microwave-based homogenized sample (coordinate axis in green) of Chinese cabbage, baby Chinese cabbage, pak choi, daikon radish (1.5 cm and 3 cm cubes) and broccoli (floret and quarterly floret) samples: (**a**) three methods, (**b**) freeze-dried sample powder and frozen-fresh sample powder and (**c**) freeze-dried sample powder and microwave-based homogenized sample. The red line represents the equivalence of *x*, *y* and *z*, while the pink shade represents that the relative standard deviation of *x* and *y* is within 20%.

In previous studies, fresh samples were frozen and ground into a fine powder with liquid nitrogen or freeze-dried and ground into a fine powder before GSL extraction [19,27]. To verify the universal applicability and accuracy of the tissue disruption methods, GSLs were extracted from Chinese cabbage, baby Chinese cabbage, pak choi, daikon radish (1.5 cm and 3 cm cubes) and broccoli (floret and quarterly floret) samples by three methods. For the convenience of expression, the GSL concentrations in the freeze-dried sample powder (μmol/g dry weight) were converted to concentrations based on fresh weight (μmol/g fresh weight) through the sample moisture content (Table S1). As shown in Figure 2, the GSL concentrations in the freeze-dried sample powder and the frozen-fresh sample powder were well fitted by the linear regression equation *y* = 0.949*x* + 0.002, with an R^2^ value of 0.998. For each vegetable, the slope ranged from 0.925 to 1.104, with R^2^ values of 0.982–1.000. These results illustrated that the two tissue disruption methods had no significant differences in the GSL extraction efficiency from different vegetables. In general, the tissue disruption method of freeze-dried samples is suitable for large plant tissues to obtain homogeneous and representative samplings, while the tissue disruption method of using frozen-fresh samples can reduce the loss of small plant tissue during processing, which is more energy-efficient and time-saving. However, there was a significant difference in GSL concentrations between the freeze-dried sample powder and the microwave-based homogenized sample. The GSL concentrations in the microwave-based homogenized sample of daikon radish (3 cm cubes) and broccoli (floret) were much lower than that in the freeze-dried sample powder, which might be caused by the retention of myrosinase activity in these samples. Microwaves are electromagnetic waves that can penetrate plant tissues and interact with polar components to generate heat. Their efficiency depends on the dissipation factor of the material and may be affected by microwave power, time and sample characteristics [12,28]. Therefore, it might not be appropriate to rely on the same microwave conditions to process different vegetable tissues. However, the microwave-based method can be used as a potentially convenient method for extracting GSLs from similar plant tissues.

**Figure 3:**
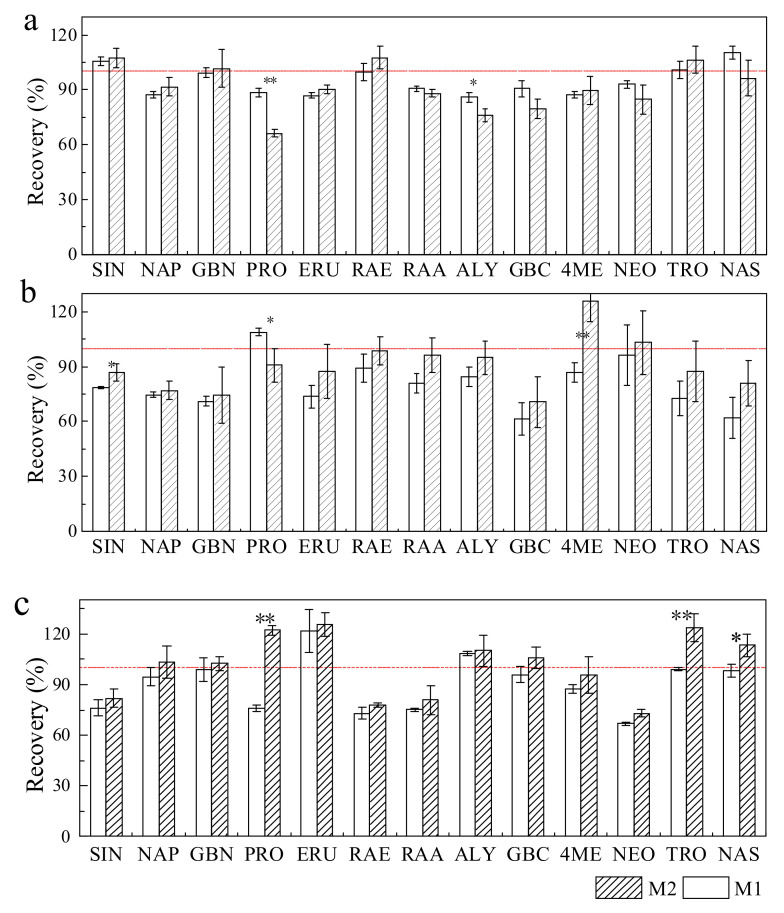
The effect of different extraction methods (M1 and M2) on the recoveries of the 13 GSLs with available standards in (**a**) the freeze-dried sample powder, (**b**) the frozen-fresh sample powder and (**c**) the microwave-based homogenized sample of Chinese cabbage. Asterisks (* and **) indicate significant differences between the two extraction methods at *p* < 0.05 and *p* < 0.01, respectively.

The extraction method was subsequently optimized by a demonstrative sample, as it influenced the extraction efficiency. In previous studies, GSLs were extracted from vegetable samples with boiling aqueous methanol, which could inactivate myrosinase [11]. However, Doheny-Adams et al. [10] found that the activity of myrosinase was inhibited by 80% methanol. Hwang et al. [15] found that the efficiency of extracting 15 GSLs from vegetable samples was slightly higher for sonication at 30 °C than for incubation at 75 °C. In this study, two different extraction methods were compared: M1 (sonication for 20 min at room temperature after vortexing the sample for 30 s) and M2 (incubation at 75 °C for 20 min and sonication for 20 min at room temperature). As shown in Figure 3, the recoveries of the 13 GSLs extracted from the freeze-dried sample powder with M1 and M2 were 86–106% and 66–108%, respectively. The progoitrin (PRO) and glucoalyssin (ALY) extraction efficiencies were significantly higher for M1 than for M2. The recoveries of the 13 GSLs extracted from the frozen-fresh sample powder with M1 and M2 were 61–109% and 74–126%, respectively. The efficiency of extracting most of the GSLs using M2 was higher than that using M1. A similar result occurred in the GSL extraction from the microwave-based homogenized sample, especially PRO. The freeze-drying and sonication procedure in methanol aqueous solution can effectively inactivate enzyme activity, which could prevent the degradation of GSLs during processing [29,30,31]. For frozen-fresh sample powder and microwave-based homogenized samples, heating is a necessary strategy for GSL extraction, which could increase the extractability of compounds in addition to inactivating the enzyme, ensuring a high GSL extraction efficiency [32].

### 2.2. Analytical Method Validation

From universality to different plant tissues, GSLs can be extracted from plants by two methods: freeze-dried sample powder of huge plant tissue is sonicated for 20 min with 70% methanol (*v*/*v*) at room temperature after vortexing the sample for 30 s, or frozen-fresh sample powder of small plant tissue is incubated with 80% methanol (*v*/*v*) at 75 °C for 20 min and sonicated for 20 min at room temperature. The extracted supernatant was diluted 10 times with ultrapure water to keep the GSL concentrations within the optimal response range, avoiding the solvent effect as the volume proportion of methanol exceeded 10%. The linearity, matrix effect, the limits of detection (LODs), the limits of quantification (LOQs), recoveries and the relative standard deviation (RSD) of the two optimal methods were validated based on UHPLC-MS/MS.

**Figure 4:**
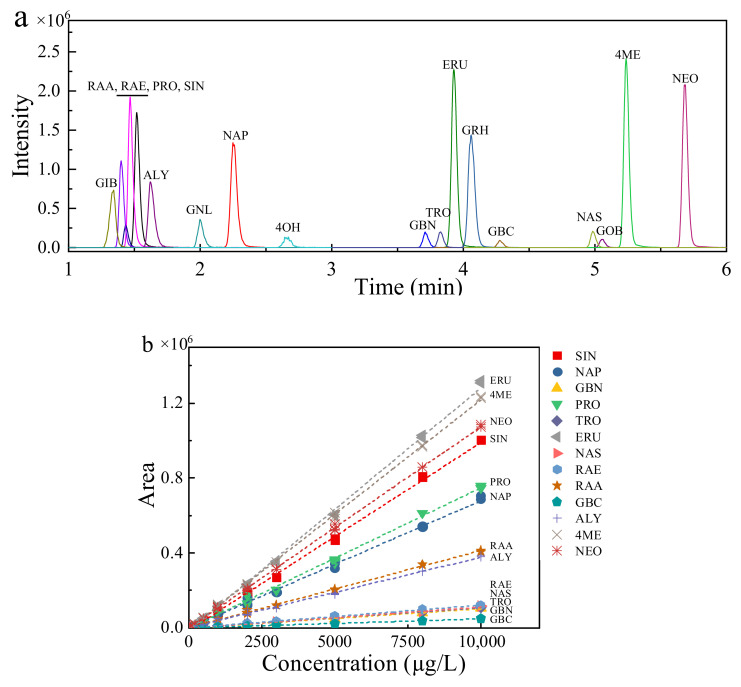
(**a**) Representative UHPLC-MS/MS chromatogram of 18 GSLs and (**b**) peak area versus concentration plots for the 13 GSLs with available standards. Note: The extracted ion chromatogram of each GSL is presented in Figure S3.

The cone voltage, collision energy and corresponding fragment ions with the best response intensity were optimized for the 13 GSLs through flow injection analysis. As shown in Table 1, the diagnostic MS/MS fragment ions of most of the GSLs were *m/z* 275, 259, 195 and 97 (their structural formulas are shown in Figure S2). These typical collision-induced fragment ions are considered characteristic of GSLs [11], which can be used as a basis for identifying GSLs without standards. In addition, the structural differences in some compounds gave rise to specific MS/MS fragment ions that could be used to distinguish isomers [27]. For example, the fragment ion at *m/z* 446 is formed via the neutral loss of a methoxy radical, which could distinguish 4-methoxyglucobrassicin (4ME) from neoglucobrassicin (NEO). The five GSLs without available standards were tentatively identified from the molecular weight, the fragment parameters reported in the literature and the vegetable species that might contain those compounds [15,27,33]. The MS/MS fragment ions of these five compounds included *m/z* 259 and 195 and their specific MS/MS fragment ions. For example, 4-hydroxyglucobrassicin (4OH) was characterized through daikon radish samples and the specific ion at *m/z* 267 via the loss of thioglucose. The GSL contents were calculated by the corresponding standard calibration curves, while those of the five GSLs without available standards were estimated using the calibration curves of structurally similar compounds. Specifically, the pair glucoberteroin (GOB) and glucoerucin (ERU), glucoiberin (GIB) and glucoraphanin (RAA), gluconapoleiferin (GNL) and PRO, and 4OH and 4ME are homologs that differ in structure by one -CH_2_ group, with glucoraphasatin (GRH) containing an alkenyl group in its molecular structure, which is two hydrogen atoms less than ERU. As shown in Figure 4, the calibration curves of 13 GSLs were well fitted by a linear regression model in the range of 10–10,000 μg/L, with R^2^ values between 0.991 and 1.000. Importantly, solvent standard curves could be used to directly quantify the GSL content in different *Brassicaceae* vegetables. This is because the coelution of matrix components did not enhance or suppress the signal responses of the target GSLs (the matrix effects were in the range of 80–120%, Figure S4) [34]. The LOQs of the 13 GSLs in the freeze-dried sample powder and frozen-fresh sample powder ranged from 5.72–17.40 nmol/g dry weight and 0.80–1.43 nmol/g fresh weight (Table 2), respectively. The accuracy and repeatability were evaluated by recoveries and RSDs using six replicates at three concentration levels. In the freeze-dried samples, the recoveries ranged from 74% to 119% with RSD less than 10%. In the frozen-fresh samples, the recoveries ranged from 77% to 104% with RSD less than 15% (Table 2). The developed method had good linearity, sensitivity, accuracy and precision and was satisfactory for detecting GSLs in vegetable samples.

### 2.3. Comparison of the UHPLC-MS/MS Method with the HPLC Method

**Figure 5:**
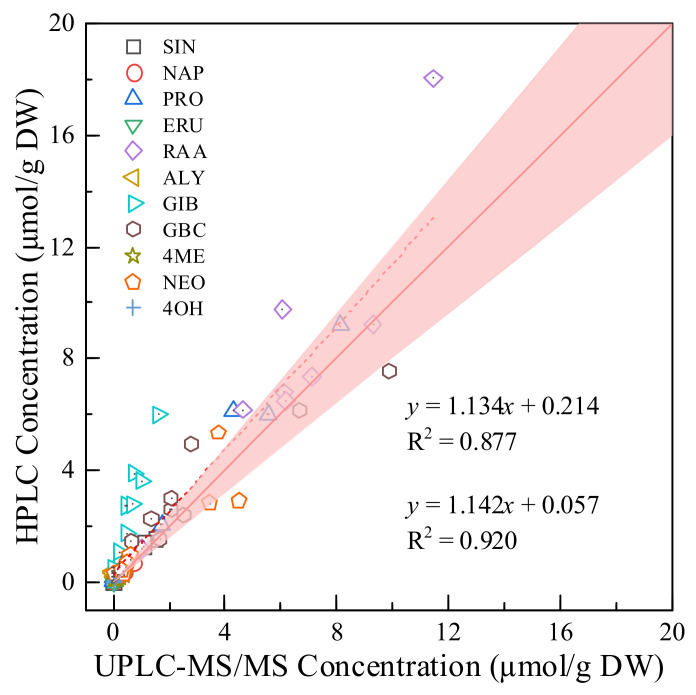
Comparison of intact GSLs analyzed by the UHPLC-MS/MS method and desulfo-GSLs analyzed by the HPLC method in broccoli. The red line represents the equivalence of *x* and *y*, while the pink shade represents that the relative standard deviation of *x* and *y* is within 20%.

GSLs are very polar because of the thioglucosyl group (−SGlc) and the strongly acidic SO_4_^2−^ residue, which must be removed (desulfation) before analysis by the most commonly used HPLC method [35,36]. The quantitative analysis method of desulfo-GSLs based on ISO 9167: 2019 was compared with the intact GSL analysis method based on UHPLC-MS/MS developed in this study. As shown in Figure 5, there was a linear relationship between the concentrations of 11 GSLs analyzed by the two methods, with the equation *y* = 1.134*x* + 0.214 and R^2^ value of 0.877. Notably, there were significant differences between the GIB concentrations analyzed by the two methods, with a slope of 3.584. The two methods were not precisely quantitative for GIB, leading to biased results. Moreover, the relationship between the results of the two methods might not be linear for single compounds, such as ALY, which might be caused by the desulfation process [20]. Not all GSLs are desulfated at the same rate; for example, glucoraphenin will be degraded or transformed during the desulfation process [10]. Incomplete desulfation, self-dimerization and self-degradation of some GSLs in the desulfation process affect the quantitative results [37]. According to Doheny-Adams et al. [10], if desulfation is required, a high concentration of sulfatase solution is required. However, the concentration of sulfatase cannot be determined when the GSL concentration in plant tissues is not clear. Moreover, a sufficient desulfurization reaction requires 15 h of incubation, which is complicated and time-consuming. Using the MRM mode, the 18 GSLs could be simultaneously quantified with excellent separation in a relatively short analysis time (Figure 4), representing only one-third of the analysis time of the HPLC method. Therefore, the UHPLC-MS/MS method established in this study is more efficient than the HPLC method in terms of operational complexity, detection speed and quantification accuracy.

### 2.4. Applications of the Developed Method

In the analysis of 15 samples, 18 GSLs belonging to three chemical classes were detected: twelve aliphatic GSLs, four indolic GSLs and two aromatic GSLs. As shown in Table S2, the detection frequencies of the aliphatic GSLs ranged from 7% to 87%, with the detection frequencies of ERU and RAA the highest. The concentration of RAA was the highest among the eight GSLs with available standards, with a maximum concentration of 17.54 μmol/g dry weight in broccoli. The detection frequencies of GOB, GIB, GRH and GNL, with no available standards, were 60%, 40%, 47% and 7%, respectively. Hwang et al. [15] reported that the detection frequencies of GOB and GIB in eight *Brassica* species were 25% and 75%, respectively. The high detection frequencies of GOB, GIB and GRH showed their wide presence in *Brassicaceae* vegetables. The detection frequencies of the four indolic GSLs were the highest, with a range of 87–100%, and the content of GBC was the highest. However, Liang et al. [27] found that the concentrations of 4ME and NEO in 12 *Brassicaceae* vegetables were significantly higher than those of GBC because GBC was used as the standard in the semiquantitative analysis of 4ME and NEO. The detection frequencies of the two aromatic compounds were 13% and 67%, respectively. The contents of aromatic compounds were lower than those of aliphatic and indolic GSLs.

**Figure 6:**
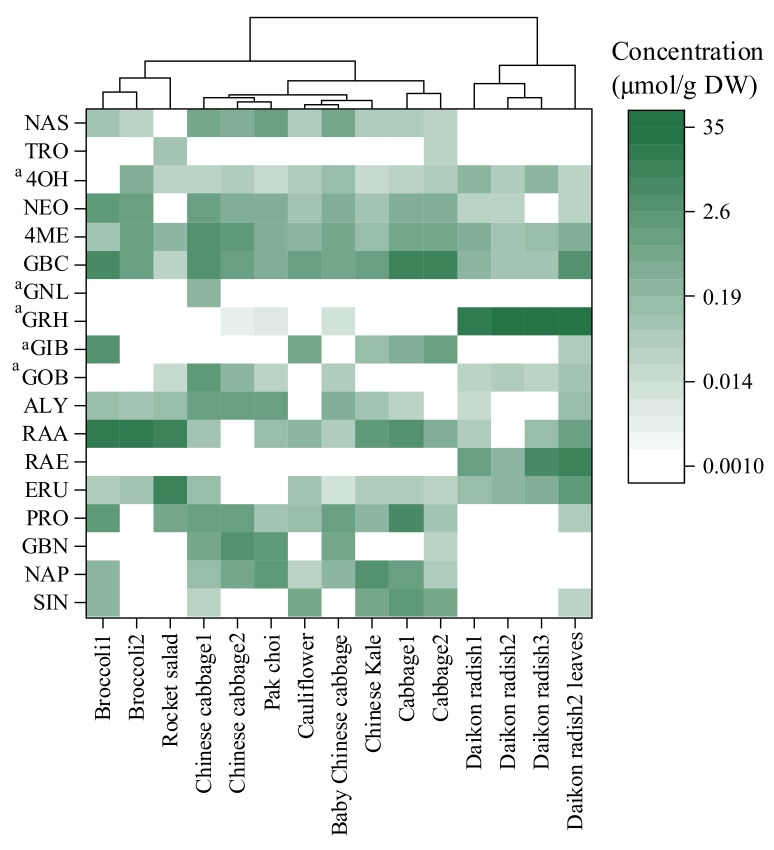
HCA of GSLs in different *Brassicaceae* vegetables. ^a^ These five GSLs have no available standards.

The contents and composition of GSLs varied significantly among different *Brassicaceae* vegetables. Aliphatic GSLs, representing 41–99% of the total GSLs, were the major components in all 15 vegetables except for the cabbage 2 sample (Figure S5). As shown in Figure 6, the highest total GSL concentration was observed in daikon radish leaves (50.28 μmol/g dry weight). The high total GSL concentrations in the daikon radish samples were due to the abundance of GRH, which accounted for approximately 66–97% of the total GSLs. In addition, RAE accounted for approximately 1–19% of the total GSLs in the daikon radish samples. GRH and RAE are the main GSLs that distinguish daikon radish from other *Brassicaceae* vegetables [27]. The concentrations of GSLs vary greatly in different plant tissues [11]. The concentrations of RAE and GBC in daikon radish leaves were significantly higher than those in daikon radish roots. Moreover, there were significant differences in GSL content among the three daikon radish samples. Li et al. [6] found substantial genotypic or intraspecific variation in GSL content among 80 broccoli genotypes, with the highest content of 12 GSLs in the florets approximately 122-fold that of the lowest genotype. Subsequently, the total GSL concentrations decreased as follows: broccoli~rocket salad~cabbage > Chinese cabbage > Chinese kale~pak choi > baby Chinese cabbage > cauliflower. RAA and GBC were the most predominant GSLs in broccoli, accounting for approximately 66% and 12% of the total GSLs, respectively. Sulforaphane, a metabolite of RAA, has been shown to inhibit the activator-protein-1 (AP-1) transcription factor and may be effective for the inhibition of ultraviolet (UV)-induced skin carcinogenesis [7]. Indole-3-carbinol is a metabolite of GBC and is a potential therapeutic for cancer prevention and treatment through WWP1-triggered PTEN reactivation [5]. Broccoli has extremely high nutritional value given the high contents of RAA and GBC. The hierarchical cluster analysis (HCA) results showed that the GSL profiles in rocket salad were similar to those in broccoli, but the content of ERU was higher and the content of GBC was lower in rocket salad than in broccoli. Indolic GSLs, accounting for 36–84% of the total GSL content, were abundant in cabbage, Chinese cabbage and baby Chinese cabbage samples. NAP was the major component in pak choi and Chinese kale, representing 28% and 40% of the total GSLs, respectively. The total GSL concentrations in baby Chinese cabbage and cauliflower were 4.93 and 3.12 μmol/g dry weight, respectively, which were much lower than those in the other *Brassicaceae* vegetables. The 15 *Brassicaceae* vegetable samples were primarily divided into three major groups, showing that the GSL profiles could be used as reference markers for the chemotaxonomic classification of *Brassicaceae* vegetables [15,27]. Most importantly, the understanding of characteristic GSLs in vegetables provides a foundation for research on functional vegetables used to meet the increasing health demands of the human diet.

## 3. Materials and Methods

### 3.1. Standards and Reagents

High-purity (>98%) GSL standards were purchased from PhytoLab GmbH & Co. KG (Vestenbergsgreuth, Middle Franconia, Germany): sinigrin (SIN), gluconapin (NAP), glucobrassicanapin (GBN), progoitrin (PRO), glucoerucin (ERU), glucoraphenin (RAE), glucoraphanin (RAA), glucobrassicin (GBC), glucotropaeolin (TRO) and gluconasturtiin (NAS). 4-Methoxyglucobrassicin (4ME) and neoglucobrassicin (NEO) were purchased from Extrasynthese (Z.I Lyon Nord, Genay Cedex France) and glucoalyssin (ALY) was purchased from Shanghai ZZBIO Co., Ltd. (Shanghai, China). HPLC-grade methanol, LC/MS-grade methanol and LC/MS-grade formic acid were supplied by Fisher Scientific (Pittsburgh, PA, USA). Ultrapure water was obtained using Milli-Q purification systems (Millipore, MA, USA). Individual stock solutions of the 13 GSLs were prepared in 70% methanol.

### 3.2. Sample Collection and Tissue Disruption

Fifteen *Brassicaceae* vegetable samples were purchased from three markets in Beijing (China), including broccoli (2), rocket salad (1), cabbage (2), cauliflower (1), pak choi (1), Chinese kale (1), Chinese cabbage (2), baby Chinese cabbage (1), daikon radish (3) and daikon radish leaves (1). Chinese cabbage was used as a demonstrative sample to optimize tissue disruption and extraction strategies. Chinese cabbage, baby Chinese cabbage, pak choi, daikon radish (1.5 cm and 3 cm cubes) and broccoli (floret and quarterly floret) samples were used to compare the extraction efficiencies of tissue disruption methods of GSLs.

Three tissue disruption methods were employed to determine optimal tissue disruption conditions. The first was to store 100 g samples at −80 °C for 24 h and then freeze-dry samples to inhibit myrosinase activity. Subsequently, the freeze-dried samples were ground into a fine powder (particle diameter < 0.425 mm) and stored at −20 °C for a maximum of three months (freeze-dried sample powder). The second one was to freeze 20 g samples in liquid nitrogen and store at −80 °C for no more than one month. Before analysis, the frozen-fresh samples were ground into a fine powder under liquid nitrogen and then extracted immediately (frozen-fresh sample powder). The third one was to process 30 g samples in a domestic microwave oven (Midea EV923KF8-NS, China) with a microwave power of 900 W for 20–180 s. The lost water was supplemented during the homogenization process at room temperature, with a 1.00 g homogenized sample immediately weighed for extraction (microwave-based homogenized sample).

### 3.3. Intact Glucosinolate Extraction

For the freeze-dried sample powder, 0.10 g of sample was placed in a 50 mL centrifuge tube (Corning Incorporated-Life Sciences, Tewksbury, MA, USA) and extracted with 10 mL of 70% methanol (*v*/*v*) containing 1000 ng/mL SIN or TRO as an internal standard. For the frozen-fresh sample powder and microwave-based homogenized sample, 1.00 g of sample was placed in a 50 mL centrifuge tube and treated with 10 mL of 80% methanol (*v*/*v*) containing 1000 ng/mL internal standard. Two methods were employed to determine the robust extraction conditions for GSLs with good extraction efficiency. The first method (M1) involved sonication for 20 min at room temperature after vortexing a sample for 30 s. The second method (M2) consisted of incubation at 75 °C for 20 min and sonication for 20 min at room temperature. Subsequently, the extracts were centrifuged at 3000× *g* for 15 min, with 1 mL of the supernatant diluted with ultrapure water to 10 mL and filtered through a 0.22 μm nylon syringe filter for UHPLC-MS/MS analysis.

### 3.4. UHPLC-MS/MS Analysis

The equipment consisted of a Waters Acquity I-class ultrahigh-performance liquid chromatography system (UHPLC) and a Xevo TQ-S micro triple quadrupole mass spectrometry (MS/MS). A Waters Acquity UPLC^®^BEH C18 column (100 × 2.1 mm, 1.7 μm particle size) maintained at 30 °C was used to separate the GSLs. The sample injection volume was 2 μL. The mobile phases consisted of ultrapure water containing 0.1% formic acid *v*/*v* (A) and methanol (B) at a flow rate of 0.2 mL/min. The gradient elution program was set as follows: 0 min, 90% A; 1.0 min, 90% A; 3.0 min, 75% A; 5.0 min, 40% A; 6.0 min, 0% A; 6.2 min, 90% A and 9.0 min, 90% A.

Tandem mass spectrometry was performed with electrospray ionization (ESI) in negative ion mode. The MS parameters were set as follows: desolvation temperature, 500 °C; capillary voltage, 2.50 kV and desolvation gas flow rate, 1000 L/h. The fragment voltage, collision energy and multiple reaction monitoring (MRM) mode transitions were optimized for each compound and were listed in Table 1.

### 3.5. Quantitative Analysis of Desulfo-Glucosinolates

Desulfo-GSLs were analyzed by HPLC based on the ISO 9167: 2019 method [36]. The freeze-dried sample powder was placed in a water bath at 75 °C for 1 min. Then, 50% boiling ethanol solution was added, with 200 μL of 20 mmol/L SIN or TRO solution added immediately as an internal standard. After heating at 75 °C for 10 min, the extracts were centrifuged at 5000× *g* for 3 min. The volume of the supernatant liquid was adjusted to 5 mL with water. Then, 0.5 mL of the extract was transferred to an ion-exchange column and rinsed with 0.02 mol/L sodium acetate solution. Seventy-five microliters of diluted purified sulfatase solution was added to the column and left to act for 15 h at room temperature. Desulfo-GSLs were eluted with ultrapure water.

HPLC analyses were performed using an LC-20A HPLC system (Shimadzu, Japan) equipped with an SPD-20AD detector. Desulfo-GSLs were separated by a Waters Nova-Pak C18 column (3.9 mm × 150 mm, 5 μm particle size) at 30 °C. The detection wavelength was 229 nm. Ultrapure water containing 0.05% tetramethylammonium chloride (A) and 0.05% tetramethylammonium chloride in 20% aqueous acetonitrile (B) were used as the mobile phase. The gradient elution program was set as follows: 0 min, 100% A; 6.0 min, 100% A; 21.0 min, 0% A; 26.0 min, 100% A and 30.0 min, 100% A. The GSL contents were calculated by the internal standard and the relative proportionality factors.

### 3.6. Quantification and Method Performance Validation

The accuracy and repeatability of the quantitative method were assessed by the recoveries and the relative standard deviation (RSD) from six replicates at three concentration levels. The sensitivity was evaluated from the limits of detection (LODs) and limits of quantification (LOQs), which were defined as signal-to-noise ratios (S/N values) of 3 and 10, respectively. The linearity was evaluated using GSL standard calibration curves. Briefly, the individual stock standard solutions were diluted with ultrapure water to prepare a series of standard solutions with a concentration range of 10–10,000 μg/L. Thirteen GSLs with available standards were quantified in samples through their corresponding calibration curves, while the remaining five GSLs (glucoberteroin (GOB), glucoiberin (GIB), glucoraphasatin (GRH), gluconapoleiferin (GNL) and 4-hydroxyglucobrassicin (4OH)), without available standards, were quantified using the calibration curves of structurally similar compounds (Table 1). The matrix effect on each GSL was evaluated by the ratio of the signal response of a spiked postextraction reference matrix sample to the same concentration in a standard solution [38].

### 3.7. Statistical Analysis

All results are presented as arithmetic means with standard errors and subjected to a one-way analysis of variance (ANOVA) or Student’s t-test using SPSS software (ver. 21, IBM, New York, NY, USA). The least significant differences were used to compare treatment means (*p* < 0.05). The GSL concentrations in each sample lower than the LODs were set to 0 for statistical analysis. Hierarchical cluster analysis (HCA) was applied to identify similarities in the GSL profiles in different *Brassicaceae* vegetables. In the HCA, the between-group linkage method as the amalgamation rule and the squared Euclidean distance as the metric were applied to establish clusters.

## 4. Conclusions

In this study, we compared the effects of different tissue disruption methods on GSL extraction. Microwave treatment was proposed as a step of plant tissue disruption to inactivate myrosinase in Chinese cabbage. However, the same microwave conditions cannot be used to process different vegetable tissues. From the universality of the method to various plant tissues, the tissue disruption method of freeze-dried samples can obtain homogeneous and representative samples that can be stored for a long time, while the tissue disruption method of frozen-fresh samples is more energy-efficient and time-saving. In addition, the extraction method of GSLs from samples with different plant tissue disruption methods was optimized and verified. Notably, the verified method of intact GSLs by UHPLC-MS/MS established in this study was more accurate and time-saving than the commonly used ISO method for desulfo-GSLs. This developed method was used to understand the characteristic GSLs in *Brassicaceae* vegetables, providing a foundation for further in-depth research on GSL-rich functional vegetables.

## Figures and Tables

**Table 1:** MRM Conditions for GSLs in UHPLC-MS/MS Analysis.

Chemical Group	Compound	Abbreviation	Chemical Formula	Retention Time (min)	Parent (*m*/*z*)	Cone (V)	Daughter (*m*/*z*)			Collision (V)		
Aliphatic	Sinigrin	SIN	C_10_H_17_NO_9_S_2_	1.52	357.86	4	96.62	161.89	194.93	18	18	20
	Gluconapin	NAP	C_11_H_19_NO_9_S_2_	2.24	371.83	4	96.56	194.81	258.94	22	16	18
	Glucobrassicanapin	GBN	C_12_H_21_NO_9_S_2_	3.69	385.91	4	194.92	258.92	274.94	16	20	20
	Progoitrin	PRO	C_11_H_19_NO_10_S_2_	1.46	387.89	4	96.75	194.91	258.91	20	18	16
	Glucoerucin	ERU	C_12_H_23_NO_9_S_3_	3.90	419.96	10	96.68	258.98	195.00	22	18	20
	Glucoraphenin	RAE	C_12_H_21_NO_10_S_3_	1.43	433.87	4	96.50	258.98	419.01	22	20	20
	Glucoraphanin	RAA	C_12_H_23_NO_10_S_3_	1.40	435.89	2	96.69	259.01	371.93	24	20	18
	Glucoalyssin	ALY	C_13_H_25_NO_10_S_3_	1.62	449.90	6	191.92	274.94	386.02	24	20	20
	Glucoberteroin *^a^*	GOB	C_13_H_24_NO_9_S_3_	5.06	434.06	10	354.11	258.98	195.03	20	20	18
	Glucoiberin *^a^*	GIB	C_11_H_21_NO_10_S_3_	1.35	422.02	12	358.00	258.98	195.00	26	15	20
	Glucoraphasatin *^a^*	GRH	C_12_H_21_NO_9_S_3_	4.08	418.03	10	338.00	258.98	175.00	18	20	18
	Gluconapoleiferin *^a^*	GNL	C_12_H_20_NO_10_S_2_	2.00	402.05	10	332.00	258.98	195.00	25	22	20
Indolic	Glucobrassicin	GBC	C_16_H_20_N_2_O_9_S_2_	4.26	446.90	18	194.97	258.95	274.94	24	18	22
	4-methoxyglucobrassicin	4ME	C_17_H_22_N_2_O_10_S_2_	5.23	476.91	10	96.62	194.93	258.90	22	18	20
	Neoglucobrassicin	NEO	C_17_H_22_N_2_O_10_S_2_	5.67	477.00	10	96.96	258.98	446.00	22	18	14
	4-hydroxyglucobrassicin *^a^*	4OH	C_16_H_20_N_2_O_10_S_2_	2.64	463.05	10	285.00	267.00	259.01	18	20	26
Aromatic	Glucotropaeolin	TRO	C_14_H_19_NO_9_S_2_	3.80	407.86	2	165.93	194.93	258.91	24	20	20
	Gluconasturtiin	NAS	C_15_H_21_NO_9_S_2_	4.96	421.91	12	179.95	194.93	258.96	22	16	20

Note: *^a^* These five GSLs have no available standards. GOB was semiquantified with the calibration curve of ERU, GIB was semiquantified with the calibration curve of RAA, GRH was semiquantified with the calibration curve of ERU, GNL was semiquantified with the calibration curve of PRO and 4OH was semiquantified with the calibration curve of 4ME.

**Table 2:** Validation of the UHPLC-MS/MS Method for 13 GSLs with Available Standards in Freeze-Dried Sample Powder and Frozen-Fresh Sample Powder.

Compound	Freeze-Dried Sample Powder					Frozen-Fresh Sample Powder					Calibration Curve Equation	R^2^
	Concentration	Recovery	RSD	LOD	LOQ	Concentration	Recovery	RSD	LOD	LOQ		
	(nmol/g DW)	(%)	(%)	(nmol/g DW)	(nmol/g DW)	(nmol/g FW)	(%)	(%)	(nmol/g FW)	(nmol/g FW)		
Sinigrin	50	107	5	3.23	9.69	5	94	1	0.37	1.11	*y* = 94.9167*x* − 810.16	0.997
	250	110	3			25	80	7				
	1000	90	5			100	90	12				
Gluconapin	50	92	6	2.52	7.56	5	91	8	0.33	0.98	*y* = 67.5645*x* − 191.872	0.993
	250	92	3			25	81	7				
	1000	77	6			100	89	3				
Glucobrassicanapin	50	99	9	2.95	8.85	5	90	14	0.34	1.03	*y* = 9.8891*x* − 75.1189	0.997
	250	99	7			25	94	2				
	1000	91	6			100	95	1				
Progoitrin	50	95	9	3.47	10.42	5	92	4	0.37	1.11	*y* = 73.4885*x* − 376.377	0.995
	250	76	4			25	90	3				
	1000	74	6			100	86	8				
Glucoerucin	50	112	4	2.11	6.34	5	82	15	0.29	0.86	*y* = 123.675*x* − 670.066	0.997
	250	100	2			25	85	4				
	1000	81	7			100	88	0				
Glucoraphenin	50	100	8	5.51	16.53	5	100	6	0.45	1.35	*y* = 12.0899*x* − 30.9665	1.000
	250	91	4			25	95	6				
	1000	77	7			100	96	1				
Glucoraphanin	50	88	6	1.91	5.72	5	103	6	0.27	0.80	*y* = 41.6557*x* − 63.9675	0.997
	250	99	3			25	98	6				
	1000	79	7			100	104	7				
Glucoalyssin	50	86	7	3.32	9.95	5	94	5	0.33	0.99	*y* = 37.7662*x* − 31.3535	0.991
	250	87	3			25	102	4				
	1000	79	6			100	101	1				
Glucobrassicin	50	119	4	5.38	16.14	5	86	2	0.44	1.32	*y* = 4.62456*x* − 33.7449	0.997
	250	92	9			25	79	13				
	1000	92	10			100	87	10				
4-Methoxyglucobrassicin	50	104	10	2.64	7.92	5	84	13	0.29	0.86	*y* = 120.093*x* − 267.184	0.999
	250	98	7			25	86	8				
	1000	79	9			100	83	2				
Neoglucobrassicin	50	109	10	2.87	8.62	5	78	6	0.30	0.90	*y* = 106.49*x* − 98.5261	0.999
	250	108	6			25	77	4				
	1000	82	8			100	80	4				
Glucotropaeolin	50	106	5	5.80	17.40	5	79	12	0.48	1.43	*y* = 10.493*x* − 62.1323	0.998
	250	101	7			25	79	4				
	1000	82	6			100	80	1				
Gluconasturtiin	50	96	5	3.60	10.79	5	78	12	0.36	1.08	*y* = 10.6905*x* − 53.0295	0.996
	250	97	1			25	77	4				
	1000	77	6			100	79	1				

Note: DW, dry weight; FW, fresh weight.

## Supplementary Materials

The following are available online: Table S1: The moisture content of 15 *Brassicaceae* vegetable samples. Table S2: Analysis of 18 GSLs in 15 *Brassicaceae* vegetable samples. Figure S1: The effect of microwave treatment at different times on the peak area of 13 GSLs in 1000 μg/L standard solution. Figure S2: Structural formula based on the MS2 fragment ions at *m/z* 275, 259, 195 and 97. Figure S3: UHPLC-MS/MS ion chromatograms showing the separation of glucosinolates analyzed in negative ion mode. Figure S4: The matrix effect of the 13 GSLs with available standards in (a) freeze-dried sample powder and (b) frozen-fresh sample powder. Figure S5: The distribution of GSLs in 15 *Brassicaceae* vegetables. GSLs in different groups are shown in different shades of the same color, e.g., aliphatic GSLs in blue, indolic GSLs in red and aromatic GSLs in green.

## Abbreviations

ALY, Glucoalyssin; DW, dry weight; ERU, Glucoerucin; FW, fresh weight; GBC, Glucobrassicin; GBN, Glucobrassicanapin; GIB, Glucoiberin; GNL, Gluconapoleiferin; GOB, Glucoberteroin; GRH, Glucoraphasatin; GSLs, Glucosinolates; HCA, Hierarchical cluster analysis; MRM, multiple reaction monitoring mode; NAP, Gluconapin; NAS, Gluconasturtiin; NEO, Neoglucobrassicin; PRO, Progoitrin; RAA, Glucoraphanin; RAE, Glucoraphenin; SIN, Sinigrin; TRO, Glucotropaeolin; 4ME, 4-Methoxyglucobrassicin; 4OH, 4-hydroxyglucobrassicin.
