# Supplementary material for: Determination of 18 Intact Glucosinolates in Brassicaceae Vegetables by UHPLC-MS/MS: Comparing Tissue Disruption Methods for Sample Preparation
Source: Molecules. 2021 Dec 30;27(1):231. doi: 10.3390/molecules27010231 (PMC8746615; doi:10.3390/molecules27010231)
Supplement: Supplementary file 1 [file molecules-27-00231-s001.zip › molecules-1507681-supplementary.pdf]

Determination of 18 intact glucosinolates in *Brassicaceae* vegetables by UHPLC-MS/MS: Comparing tissue disruption methods for sample preparation

Xiaolu Yu, Hongju He, Xuezhi Zhao, Guangmin Liu, Liping Hu, Bing Cheng, Yaqin Wang*

Institute of Agri-food Processing and Nutrition, Beijing Academy of Agriculture and Forestry Sciences, Beijing 100097, China

Beijing Vegetable Research Center, Beijing Academy of Agriculture and Forestry Sciences, Beijing 100097, China

***Corresponding author:** Yaqin Wang. Email: wangyaqin@iapn.org.cn

Table S1 The moisture content of 15 *Brassicaceae* vegetable samples.

Samples	Moisture content (%)
Broccoli 1	90.98
Broccoli 2	89.54
Rocket salad	89.50
Cabbage 1	93.25
Cabbage 2	92.18
Cauliflower	91.02
Pak choi	91.61
Chinese kale	90.20
Chinese cabbage 1	91.57
Chinese cabbage 2	92.01
Baby Chinese cabbage	92.20
Daikon radish 1	95.09
Daikon radish 2	94.00
Daikon radish 3	94.54
Daikon radish leaves	89.96

Table S2 Analysis of 18 GSLs in 15 *Brassicaceae* vegetable samples.

Compound	Abbreviation	Detection Frequency (%)	Concentration (μmol/g DW)			
			Average	Median	Maximum	Minimum
Sinigrin	SIN	47	0.29	ND	1.87	ND
Gluconapin	NAP	60	0.56	0.039	3.19	ND
Glucobrassicanapin	GBN	33	0.38	ND	2.84	ND
Progoitrin	PRO	73	0.77	0.099	4.82	ND
Glucoerucin	ERU	87	0.90	0.081	10.07	ND
Glucoraphenin	RAE	27	1.02	ND	9.76	ND
Glucoraphanin	RAA	87	3.11	0.20	17.54	ND
Glucoalyssin	ALY	73	0.34	0.078	1.40	ND
Glucoberteroin[a]	GOB[a]	60	0.16	0.030	1.72	ND
Glucoiberin[a]	GIB[a]	40	0.36	ND	3.23	ND
Glucoraphasatin[a]	GRH[a]	47	6.84	ND	33.00	ND
Gluconapoleiferin[a]	GNL[a]	7	0.013	ND	0.19	ND
Glucobrassicin	GBC	100	2.46	1.09	11.76	0.038
4-Methoxyglucobrassicin	4ME	100	0.69	0.44	3.12	0.092
Neoglucobrassicin	NEO	87	0.46	0.21	2.36	ND
4Hydroxyglucobrasscin[a]	4OH[a]	93	0.089	0.042	0.36	ND
Glucotropaeolin	TRO	13	0.0080	ND	0.080	ND
Gluconasturtiin	NAS	67	0.22	0.039	0.98	ND

Note: [a]These five glucosinolates have no available standards.

ND means not detected.

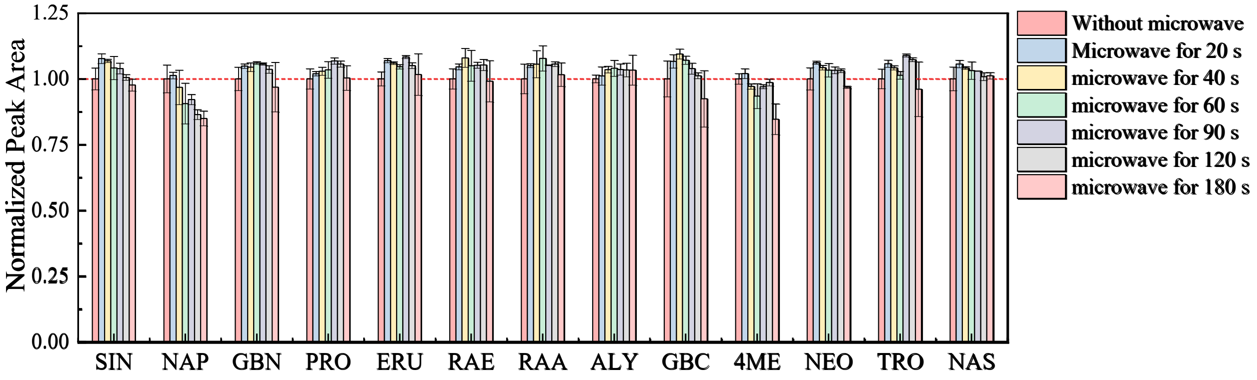

Figure S1 The effect of microwave treatment at different times on the peak area of 13 GSLs in 1000 μg/L standard solution.

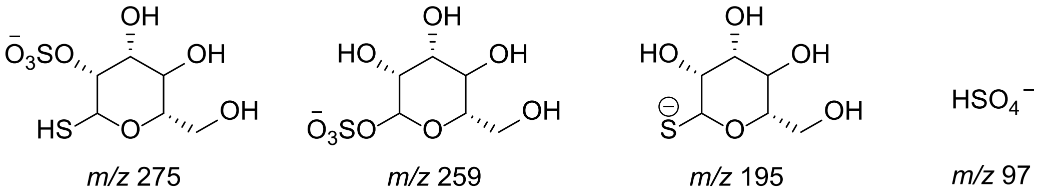

Figure S2 Structural formula based on the MS/MS fragment ions at *m/z* 275, 259, 195 and 97.

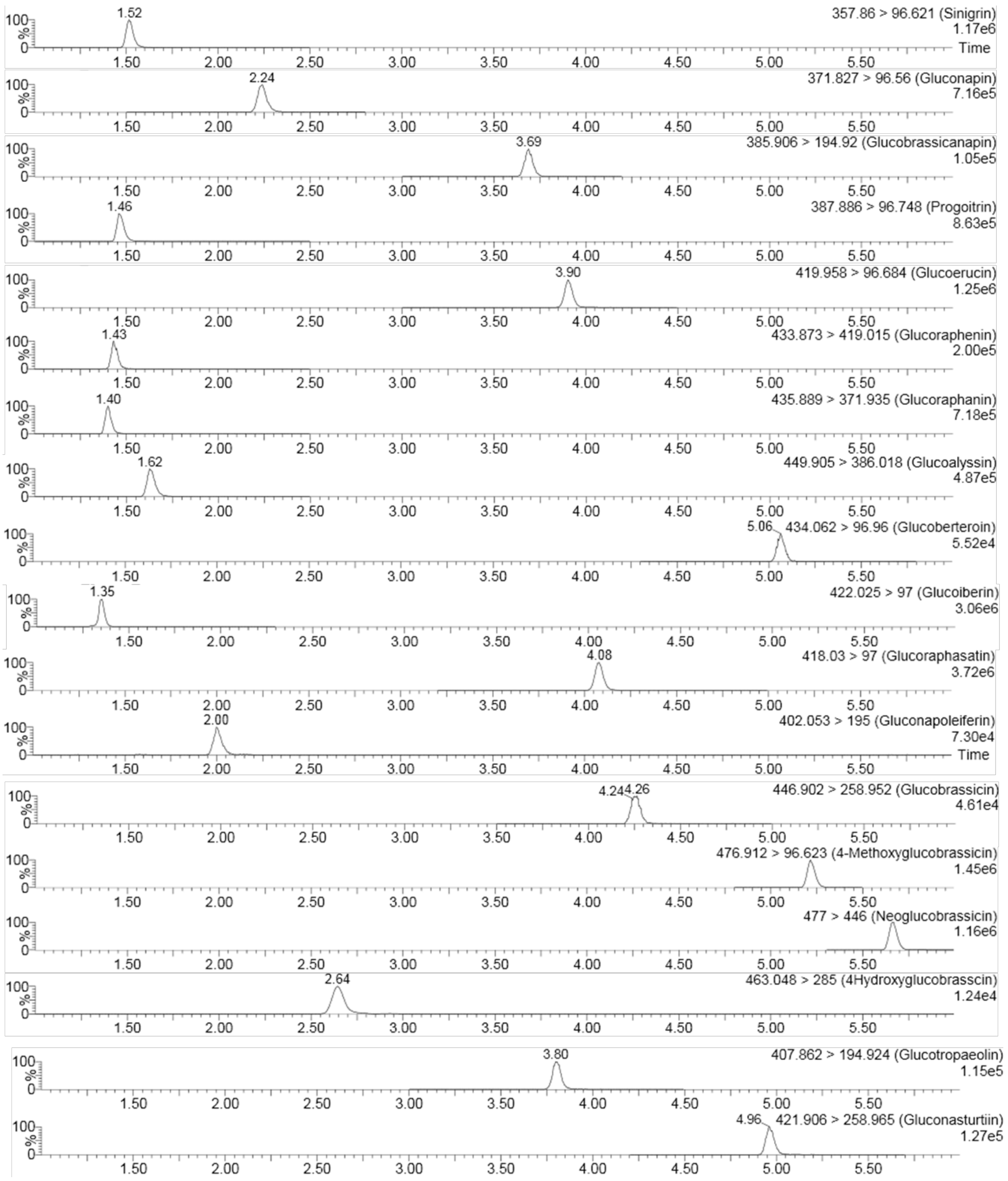

Figure S3 UHPLC-MS/MS ion chromatograms showing the separation of glucosinolates analyzed in negative ion mode.

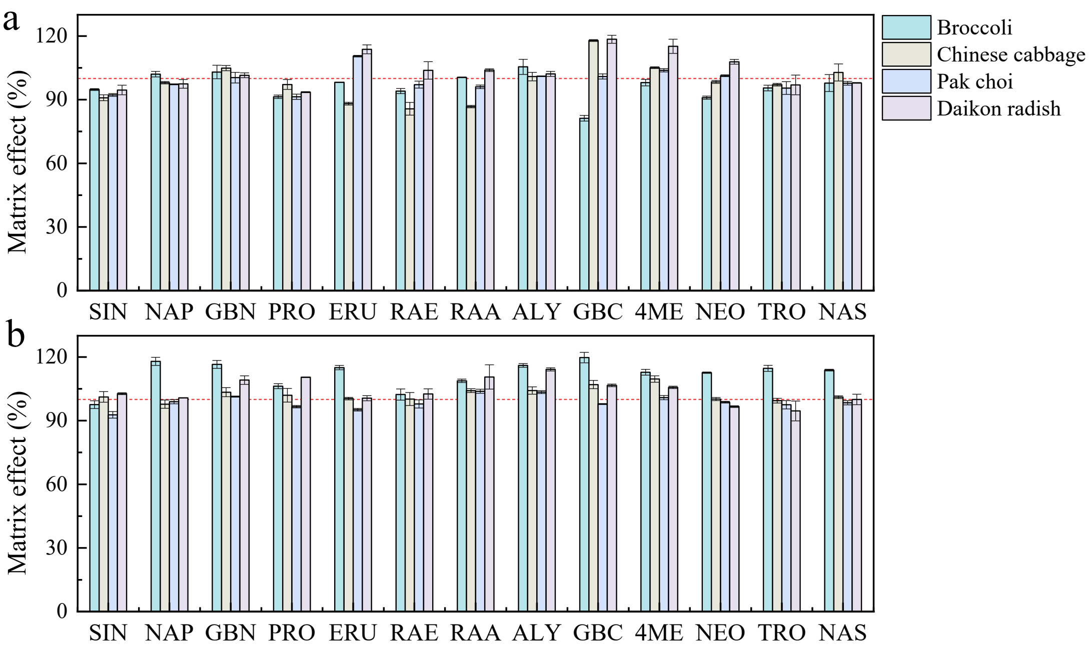

Figure S4 The matrix effect of the 13 GSLs with available standards in (a) freeze-dried sample powder and (b) frozen-fresh sample powder.

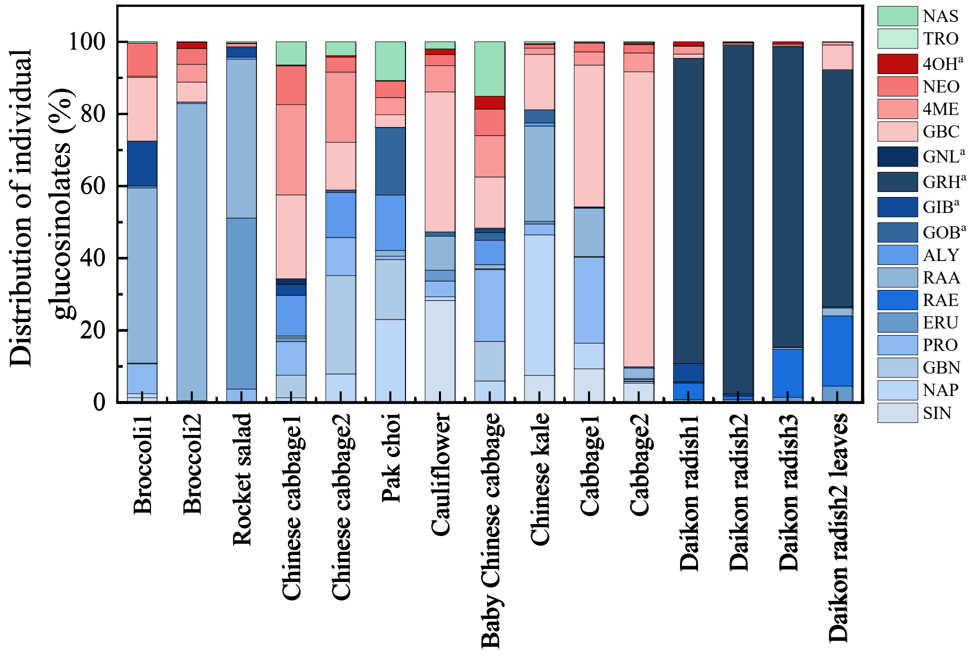

Figure S5 The distribution of GSLs in 15 *Brassicaceae* vegetables. GSLs in different groups are shown in different shades of the same color, e.g., aliphatic GSLs in blue, indolic GSLs in red, and aromatic GSLs in green.
